# Ultrasound-based liver elastography: a narrative review of technical principles and interpretation of results

**DOI:** 10.1590/0100-3984.2025.0102

**Published:** 2026-04-23

**Authors:** Fernanda Fernandes Souza, Roberta Chaves Araújo, Guilherme Massotte Fontanin, Jorge Elias Júnior

**Affiliations:** 1 Department of Internal Medicine, School of Medicine, University of São Paulo at Ribeirão Preto, Ribeirão Preto, SP, Brazil.; 2 Clinical Hospital, School of Medicine, University of São Paulo at Ribeirão Preto, Ribeirão Preto, SP, Brazil.; 3 Department of Medical Imaging, Hematology and Clinical Oncology, School of Medicine, University of São Paulo at Ribeirão Preto, Ribeirão Preto, SP, Brazil.

**Keywords:** Elasticity imaging techniques/standards, Ultrasonography, Liver cirrhosis/diagnostic imaging, Liver diseases/diagnostic imaging, Técnicas de Imagem por Elasticidade/normas, Ultrassonografia, Cirrose hepática/diagnóstico por imagem, Hepatopatias/diagnóstico por imagem

## Abstract

In recent years, noninvasive methods for assessing liver fibrosis have significantly transformed hepatology practices, reducing the need for liver biopsy. Among these, elastography techniques have become widely used tools for staging fibrosis in patients with chronic liver disease. Currently, various modalities are available, including transient elastography, point shear wave elastography, two-dimensional shear wave elastography, and magnetic resonance elastography; the first three are ultrasound-based techniques, whereas the last is a magnetic resonance imaging-based technique. In this context, ultrasound-based liver elastography stands out as a reliable, reproducible method with high accuracy. This narrative review aims to present the technical principles of ultrasound-based shear wave elastography, discuss the factors that influence measurement reliability, and provide practical guidance for result interpretation. The topics addressed include acquisition protocols, methodological limitations, potential confounding factors, and clinically relevant cutoff values. In conclusion, when applied under appropriate indications with adequate technical quality and interpretation, ultrasound-based liver elastography is a valuable, complementary tool for managing chronic liver disease.

## Abstract (pt)

Nos últimos anos, métodos não invasivos de avaliação da fibrose hepática transformaram significativamente a prática da hepatologia, reduzindo a necessidade de biópsias hepáticas. Entre eles, as técnicas de elastografia se consolidaram como ferramentas amplamente utilizadas para o estadiamento da fibrose em pacientes com doença hepática crônica. Atualmente, diferentes modalidades estão disponíveis, incluindo a elastografia transitória, a elastografia por ondas de cisalhamento pontual, a elastografia bidimensional e a elastografia por ressonância magnética - as três primeiras são técnicas baseadas em ultrassom, enquanto a última é baseada em ressonância magnética. Nesse contexto, a elastografia hepática por ultrassonografia destaca-se por ser uma técnica acessível, reprodutível e com boa acurácia. Esta revisão narrativa tem por objetivo apresentar os princípios técnicos da elastografia por ondas de cisalhamento, discutir fatores que influenciam a confiabilidade das medidas e oferecer orientações práticas para a interpretação dos resultados. São abordados aspectos relacionados ao protocolo de aquisição, limitações do método, potenciais fatores de confusão, bem como valores de corte de relevância clínica. Conclui-se que, quando aplicada em condições ideais de indicação, execução e interpretação, a elastografia hepática por ultrassonografia constitui uma ferramenta útil e complementar no manejo da doença hepática crônica.

## INTRODUCTION

Chronic liver disorders represent a significant public health concern worldwide, accounting for more than two million deaths each year and approximately 4% of all deaths worldwide, ranking as the eleventh leading cause of mortality ^**(1)**^. Most liver-related deaths result from complications of cirrhosis and hepatocellular carcinoma, conditions largely driven by chronic viral hepatitis, alcohol-related liver disease, and metabolic dysfunction-associated steatotic liver disease (MASLD). Liver disease predominantly affects young and middleaged adults, leading to a significant number of potential years of life lost. In addition, its economic and healthcare implications are escalating on a global scale.

Chronic liver diseases are typically silent for many years, with patients often remaining asymptomatic until the condition advances to a more severe stage. Natural history underscores the critical importance of devising effective strategies for early detection, which facilitates timely intervention and enables appropriate treatments. Early diagnosis helps prevent or delay progression to cirrhosis and its complications, including portal hypertension, hepatic decompensation, and hepatocellular carcinoma. The advent of liver elastography represents a significant advancement in the noninvasive assessment of liver fibrosis. Key benefits of elastography techniques include providing quantitative information on liver stiffness, enabling early recognition of liver fibrosis or cirrhosis prior to the onset of symptoms or complications, as well as reducing the need for invasive liver biopsies. Although biopsy is traditionally considered the gold standard, it is limited by its invasive nature and potential for sampling variability and complications. Consequently, elastography has emerged as a valuable tool for screening, monitoring, and guiding therapeutic decisions in patients with chronic liver disease.

Several elastography techniques are currently available, each with specific principles and applications. Transient elastography was the first to be widely adopted, demonstrating the feasibility of noninvasive liver stiffness measurement in large populations^**(2)**^. Subsequently, ultrasound-based shear wave elastography (SWE) methods, including acoustic radiation force impulse (ARFI), expanded clinical applicability by allowing point or regional assessment during conventional ultrasound examinations^**(3,^4^)**^. More recently, magnetic resonance elastography has emerged as the most comprehensive technique, offering whole-liver evaluation with high reproducibility, albeit at a higher cost and with limited availability^**(5)**^. Together, these modalities have consolidated elastography as an important part of noninvasive liver assessment^**(6)**^.

### Technical principles of liver elastography

Although mechanical changes in the liver resulting from fibrotic deposition increase tissue stiffness, fibrosis itself cannot be qualitatively characterized on physical examination. Physical examination findings such as hepatomegaly, a nodular liver surface, splenomegaly, ascites, and stigmata of chronic liver disease are indirect manifestations and typically appear only in advanced stages, such as established cirrhosis and its complications. In this context, elastography techniques have emerged as complementary imaging methods that can quantitatively assess the biomechanical properties of tissues resisting shear deformation and related to their restoring forces^**(6,^7^)**^. Shear waves are generated when a directional force is applied to tissue, causing it to deform. This force can be induced by compression or vibration at the body surface, as well as by physiological motion, or can be generated electronically through an ultrasound transducer that produces acoustic radiation force at a specific depth^**(6)**^.

Tissue stiffness is directly related to the propagation velocity of shear waves, which is the most frequently measured physical parameter in elastography^**(7)**^. This velocity can be converted into kilopascals, corresponding to the Young’s modulus, by using the formula *E* = 3 *v*^2^ (where is the tissue density and *v* is the shear wave velocity), under the assumption that the tissue is purely elastic, is incompressible, is linearly elastic, and has a density of approximately 1,000 kg/m^3**(8)**^. The SWE modality encompasses ultrasound-based techniques that evaluate liver stiffness by generating shear waves through a mechanical push (external or internal). In clinical practice, SWE methods integrated into ultrasound systems rely on an internal acoustic push pulse created by an ARFI to induce shear waves directly within the liver parenchyma. Tissue elasticity is quantified by measuring the shear wave speed. This can be performed at a single location, as in point SWE (pSWE), or by combining multiple ARFI lines to generate quantitative elasticity maps, as achieved with the two-dimensional SWE (2D-SWE) technique^**(6,^9^)**^.

Considering its growing role in clinical practice and research, this narrative review focuses on ultrasound-based SWE methods, discussing their technical principles and interpretation of results.

### pSWE

**Figure 1.**
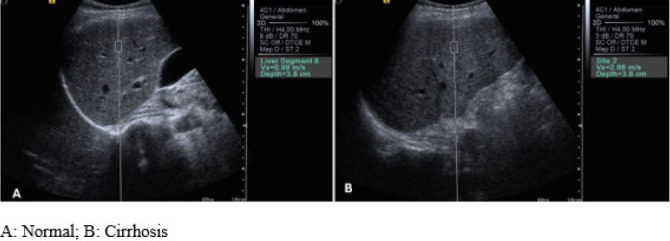
Example of ultrasound-based liver elastography using the pSWE technique. **A**, normal; **B**, cirrhosis.

The first method to integrate specialized software into standard ultrasound equipment for elastography was pSWE. The B-mode image provides real-time liver visualization, allowing the selection of the region of interest (ROI) for shear wave velocity measurement^**(6)**^. The operator can position the rectangular ROI box, approximately 10 × 6 mm, within the target area, as shown in Figure 1. Once activated, the transducer emits short acoustic pulses that generate shear waves in the ROI. Tracking pulses then detects these waves, quantifying tissue stiffness in meters per second or kilopascals, depending on the device, directly reflecting the elasticity of the hepatic parenchyma^**(6)**^.

### 2D-SWE

**Figure 2.**
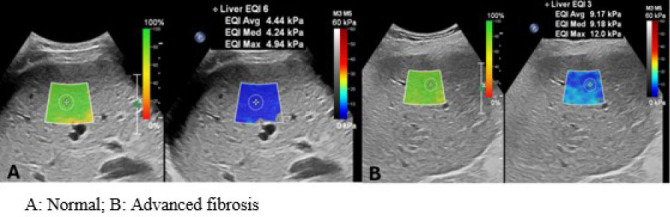
Example of ultrasound-based liver elastography using the 2D-SWE technique. **A**, normal; **B**, advanced fibrosis.

Based on the same physical principles, this technique generates a two-dimensional quantitative stiffness map. In this approach, shear waves are induced at progressively increasing depths within the tissue at an ultrafast speed, propagating throughout the entire imaging field. This enables the real-time assessment of shear wave velocity and the generation of elastograms that represent the Young’s modulus of the tissue, expressed in either kilopascals or meters per second. On 2D-SWE, elastographic maps are displayed over an extended two-dimensional ROI, in either a color scale or a gray scale, and can be presented independently or side by side with the B-mode image^**(6,^7^,^10^)**^, as shown in Figure 2.

### Recommended protocol for liver stiffness measurement in ARFI-based ultrasound

Several technical and patient-related factors should be standardized for liver stiffness measurement (LSM) with ARFI-based ultrasound techniques. Patients are advised to fast for at least four hours before the examination. Measurements should be performed in the right lobe of the liver, through an intercostal space, with the patient in the supine or slight left lateral position and the right arm in maximal extension. A minimum resting period of 10 min is recommended prior to the examination. The transducer should be positioned perpendicular to the liver capsule, avoiding rib shadows, large vessels, and bile ducts. The ROI should be positioned in an area of uniform parenchyma, typically located 15–20 mm below the liver capsule, where shear wave generation is most effective, typically with a distance of 4.0–4.5 cm between the transducer and the liver capsule. Examinations should be carried out during a short breath hold and neutral breathing to minimize motion artifacts. In pSWE, a set of 10 measurements should be acquired, whereas in 2D-SWE, five measurements are generally sufficient when the manufacturer’s quality metrics are available. All measurements should be independent acquisitions conducted at the same site, with each acquisition performed during a separate breath hold. Results must be reported as the median value, together with the interquartile range-to-median (IQR/M) ratio, which serves as a measure of reliability. The main reliability threshold is an IQR/M ratio ≤ 30% for stiffness values expressed in kilopascals and ≤ 15% for velocity values expressed in meters per second. At least 60% of the measurements should be considered valid. Measurements in the left liver lobe should be avoided because they tend to show higher variability. Known confounding factors that may lead to overestimation of stiffness include severe hepatic inflammation, cholestasis, hepatic congestion, acute hepatitis, and infiltrative liver disease^**(7,^10^–^12^)**^. The key technical and reliability criteria for LSM with ARFI-based ultrasound are outlined in Chart 1.

**Chart 1:** Recommendations for reliable acquisition of LSMs using ultrasound-based SWE.

Category	Recommendation
Patient preparation	Fast for at least 4 h before the examination; rest for at least 10 min prior to the examination
Patient positioning	Supine or slightly left (≈ 30°) lateral position with the right arm in maximal extension
Probe positioning	Intercostal approach; transducer perpendicular to the liver capsule, avoiding ribs, vessels, and bile ducts
ROI placement	Homogeneous parenchyma, 15–20 mm below the capsule; optimal depth 4.0–4.5 cm from the transducer; each measurement obtained from a distinct image, in the same location
Breathing	Neutral breathing (avoiding deep inspiration) and breath hold
Number of measurements	pSWE: 10 measurements; 2D-SWE: 5 measurements
Reporting	Median value and IQR/M ratio (in kPa or m/s)
Reliability criteria	IQR/M ratio ≤ 30% (kPa) or ≤ 15% (m/s); ≥ 60% valid acquisitions
Preferred site	Right liver lobe (left lobe measurements discouraged because of variability)
Confounding factors	Severe inflammation (AST/ALT > 5× ULN), cholestasis, congestion, acute hepatitis, and infiltrative disease

AST, aspartate aminotransferase; ALT, alanine aminotransferase, ULN, upper limit of normal.

It should be borne in mind that elastography is a technology that is continually evolving. Although several devices based on similar principles are available in the market, they exhibit differences in some aspects, such as image acquisition, data processing, and sampling rates. Consequently, a thorough examination of each manufacturer’s instructions and validation studies published in clinical settings is advised. In addition, new systems and software are frequently introduced, and updates are made to enhance and refine the performance of the existing equipment.

### Factors other than liver fibrosis that influence liver stiffness

Several factors unrelated to fibrosis may influence liver stiffness values obtained by elastography, acting as potential confounders in the clinical interpretation of these values. These include hepatic inflammation (due to conditions such as acute hepatitis or transaminase flares), cholestasis, congestive heart failure, and infiltrative liver disease. Other transient conditions, such as recent food intake, vigorous physical activity, and deep inspiration, should also be considered^**(6,^13^)**^.

It is important to note that histological evaluation and SWE do not assess the same parameters. Histology provides a detailed analysis of distinct features, such as fibrosis, steatosis, inflammation, iron deposition, and, in the context of liver transplantation, criteria for rejection. In contrast, SWE estimates liver stiffness, which primarily reflects the degree of fibrosis but may also be affected by confounding factors, such as inflammation or congestion. In addition, ultrasound attenuation-based fat quantification techniques have been developed, although that topic is beyond the scope of the present review. Therefore, if elastography results do not align with the expected clinical or histological context, potential confounding factors must be rigorously evaluated and incorporated into the interpretative process.

### Interpretation of results

The interpretation of an LSM requires careful integration with clinical, laboratory, and imaging data, as well as consideration of the technical conditions during acquisition. Although elastography provides a noninvasive estimate of fibrosis, the results should never be interpreted in isolation.

From a clinical perspective, fibrosis staging is crucial for identifying patients with advanced fibrosis/cirrhosis who, even after treatment of the etiology (e.g., chronic hepatitis C, chronic hepatitis B, alcoholic liver cirrhosis), require ongoing screening programs for hepatocellular carcinoma and portal hypertension. In this context, the concept of compensated advanced chronic liver disease (cACLD), introduced by the Baveno VI consensus^**(14)**^, reflects the recognition that severe fibrosis and cirrhosis represent a continuum in asymptomatic individuals, in whom a clear clinical distinction between stages is often not feasible. Progression to clinically significant portal hypertension (CSPH) is an important point for predicting outcomes. The defining characteristic of CSPH is a hepatic venous pressure gradient ≥ 10 mmHg, which is consistently associated with an increased risk of decompensation, variceal bleeding, other liver-related events, and mortality^**(14,^15^)**^. Consequently, noninvasive tests, such as LSM by ultrasound-based elastography, have been increasingly explored as surrogate markers to identify both cACLD and CSPH, thereby improving risk stratification and guiding surveillance strategies. In this context, the 2020 revision of the Society of Radiologists in Ultrasound consensus introduced the so-called “rule of four” for SWE, proposing a simplified framework to stratify the risk of advanced chronic liver disease across different etiologies, including viral hepatitis and MASLD^**(13)**^. The 2024 World Federation for Ultrasound in Medicine and Biology Guideline/Guidance on Liver Multiparametric Ultrasound subsequently endorsed the applicability of this framework to ARFI-based SWE techniques^**(16)**^, emphasizing that it may be used to estimate the risk of advanced disease independently of the underlying etiology, provided that confounding factors affecting liver stiffness measurement are confidently excluded. According to this updated interpretation^**(16)**^, ARFI-SWE values ≤ 5 kPa (≈ 1.3 m/s) have a high probability of representing a normal liver; values < 9 kPa (≈ 1.7 m/s) rule out cACLD unless clinical signs suggest otherwise, in which case complementary testing is recommended; values of 9–13 kPa (≈ 1.7–2.1 m/s) are suggestive of cACLD but require confirmatory evaluation; values > 13 kPa (≈ 2.1 m/s) rule in cACLD; values > 17 kPa (≈ 2.4 m/s) are suggestive of CSPH; and values > 21 kPa (≈ 2.6 m/s) indicate a high probability of CSPH.

### Limitations

Despite its widespread clinical application, SWE has important limitations. Technical failures may occur in patients with obesity or unfavorable anatomical conditions, which can restrict the feasibility and accuracy of measurements. In addition, variability persists among devices and manufacturers, despite ongoing efforts toward standardization. The SWE results are also influenced by operator expertise, because the technique requires appropriate training and involves a learning curve that may affect reproducibility across examiners.

When these limitations are present, they should be consistently reported in the clinical record to minimize the risk of misinterpretation.

### Essential information in ultrasound-based elastography reports

**Figure 3.**
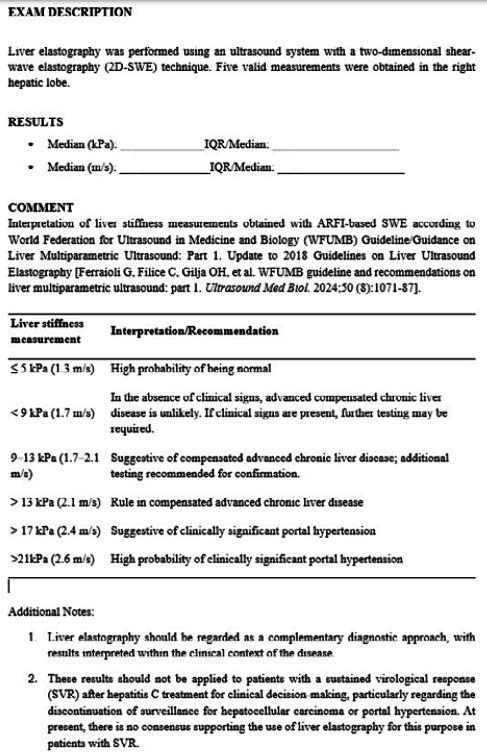
Explanatory notestohighlightpotential limitations, technical issues, or clinical conditions thatmayinfluence stiffnessmeasurements.

To ensure standardization and clarity in reporting, the elastography report should provide key technical details, including the ultrasound system used, the type of transducer employed, measurement depth, the number of valid acquisitions, and whether liver stiffness values are expressed in kilopascals or meters per second. The IQR/M ratio must also be documented, because it serves as an indicator of the measurement reliability^**(10)**^. Interpretation should be guided by established cutoff values, such as the “rule of four” for patients with viral hepatitis and MASLD^**(13,^16^)**^. When appropriate, explanatory notes are encouraged to highlight potential limitations, technical issues, or clinical conditions that may influence stiffness measurements. An illustrative example is provided in Figure 3.

### Future directions

Elastography has significantly advanced the field of hepatology by reducing the need for liver biopsy, informing therapeutic decisions, and facilitating non-invasive longitudinal monitoring. Modern ultrasound systems now enable multiparametric evaluation of the liver; in addition to elastography, these systems can quantify liver fat by measuring the ultrasound attenuation coefficient, offering valuable information for patient follow-up and prognosis. Concurrently, MASLD has reached concerning epidemiological levels, affecting nearly 38% of adults worldwide, with projections surpassing 55% by 2040, and exhibits the highest prevalence in Latin America^**(17)**^. In this context, a recent global Delphi consensus introduced an etiologically driven classification, diagnosing MASLD when hepatic steatosis is seen in conjunction with at least one cardiometabolic risk factor and no competing predominant etiology^**(18)**^. The natural history of MASLD spans simple steatosis, steatohepatitis, fibrosis, cirrhosis, and hepatocellular carcinoma, with fibrosis stage being the strongest predictor of liver-related mortality^**(19,^20^)**^. In addition, cardiovascular disease and extrahepatic cancers are major causes of death even in the absence of cirrhosis^**(19,^21^)**^. Supporting the systemic nature of the disease, Deng et al.^**(22)**^ demonstrated that hepatic fat fraction, quantified by determining the magnetic resonance imaging proton density fat fraction, is associated with an increased risk of diabetes in individuals with obesity. Furthermore, the Brazilian Diabetes Society has emphasized the importance of MASLD screening in patients with prediabetes or type 2 diabetes mellitus^**(23)**^.

Finally, noninvasive tools have proven effective in excluding advanced fibrosis and predicting liver-related events. Composite algorithms that integrate serum biomarkers with imaging-based technologies have demonstrated strong prognostic performance^**(24)**^. Looking ahead, advances in multiparametric imaging, quantitative biomarkers, and artificial intelligence-assisted work-flows are expected to further enhance the noninvasive assessment of liver disease.

## CONCLUSION

Ultrasound-based liver elastography is a useful tool in the management of chronic liver disease, particularly when applied under optimal conditions, including appropriate clinical indications, high technical quality, performance by experienced operators, and careful consideration of its limitations. Results should always be interpreted within a broader clinical and laboratory context. When elastography findings are inconsistent with the clinical scenario, the examination should be repeated. If the discordance remains unexplained, other methods for assessing liver fibrosis should be recommended as reference standards. Thus, elastography can represent a safe and accessible noninvasive method that should be integrated into a multiparametric approach to optimize its clinical applicability.

## Data Availability

Not applicable
